# The Effectiveness of Automatic Recommending System for Premedication in Reducing Recurrent Radiocontrast Media Hypersensitivity Reactions

**DOI:** 10.1371/journal.pone.0066014

**Published:** 2013-06-19

**Authors:** Yun-Jeong Bae, Ye Won Hwang, Sun-young Yoon, Sujeong Kim, Taehoon Lee, Yoon Su Lee, Hyouk-Soo Kwon, You Sook Cho, Myung Jin Shin, Hee-Bom Moon, Tae-Bum Kim

**Affiliations:** 1 Division of Allergy and Clinical Immunology, Department of Internal Medicine, Asan Medical Center, University of Ulsan College of Medicine, Seoul, Korea; 2 Department of Health Medicine, Health Screening and Promotion Center, Asan Medical Center, Seoul, Korea; 3 Pharmacovigilance Center, Asan Medical Center, Seoul, Korea; 4 Department of Radiology, Asan Medical Center, University of Ulsan College of Medicine, Seoul, Korea; University of Louisville, United States of America

## Abstract

**Background:**

Non-ionic radiocontrast media (RCM) is rarely associated with hypersensitivity reactions. Premedication of patients who reacted previously to RCM with systemic corticosteroids and/or antihistamines can help reduce recurrent hypersensitivity reactions. However, premedication is still not prescribed in many cases for various reasons. This study aimed to determine the effectiveness of our novel RCM hypersensitivity surveillance and automatic recommending system for premedication.

**Methods and Results:**

Hospitalized patients with a history of RCM hypersensitivity were identified in an electronic medical record system that included a mandatory reporting system for past adverse drug reactions. In 2009, a novel automatic prescription system was added that classified index RCM reactions by severity and dispensed appropriate corticosteroid and/or antihistamine pretreatment prior to new RCM exposures. The data from 12 months under the previous system and 12 months under the current system were compared.

The two systems had similar overall premedication rates (91% and 95%) but the current system was associated with a significantly higher corticosteroid premedication rate (65% *vs.* 14%), which significantly reduced the breakthrough reaction rate (6.7% *vs.* 15.2%). The current system was also associated with increased corticosteroid and antihistamine premedication of patients with a mild index reaction (61% *vs.* 7%) and a reduction in their breakthrough reaction rate (6% *vs.* 15%).

**Conclusions:**

Premedication with corticosteroid and/or antihistamine, which was increased by our novel automatic prescription system, significantly reduced breakthrough reactions in patients with a history of RCM hypersensitivity.

## Introduction

Radiocontrast media (RCM) are highly useful, as indicated by their frequent use in imaging departments all over the world. Indeed, more than 75 million iodinated RCM administrations are made each year worldwide [1], which may be an underestimation because the numerous procedures that use RCM, for the purpose of health screening and early diagnosis of a disease, are rapidly increasing, resulting in increasing adverse reactions to RCM. Although RCM is remarkably well tolerated in general, there are occasionally hypersensitivity reactions to RCM, which can be divided into immediate (0.5–3%) and nonimmediate reactions [2]. In particular, it is well known that patients with a history of previous RCM hypersensitivity reactions or an allergic diathesis are at increased risk of developing a hypersensitivity reaction (7.4 and 4.1%, respectively) compared to patients without these risk factors (1.2%) [3], [4].

Considering the high frequency of RCM applications, it is important that hypersensitivity reactions are stringently controlled. Patients at high risk of an immediate RCM reaction are often premedicated with systemic corticosteroid and antihistamines to prevent such reactions during subsequent RCM-based imaging analyses [5]. However, the effectiveness of premedication in terms of lessening the likelihood of RCM hypersensitivity reactions in patients with a history of these reactions remains poorly defined, although a recent study from Korea showed that premedication of patients with a history of severe RCM reactions with corticosteroids and H1 antihistamines and/or H2 blockers effectively prevented most RCM hypersensitivity reactions [6]. It remains unclear whether premedication should be used in cases where the previous RCM hypersensitivity reaction was mild.

At present, many general hospitals use a variety of computerised reporting systems that are based on the voluntary notification of adverse drug reactions [7], [8]. In our previous study, we described a mandatory reporting system for past drug hypersensitivity reactions (DHRs) that is supervised by allergy specialists in our general hospital; this system was found to be very effective in improving the management of patients with a history of drug hypersensitivity and preventing drug hypersensitivity reactions [9]. To manage RCM hypersensitivity reactions in hospitalised patients as effectively as possible, we have modified our electronic medical record (EMR)-based adverse drug reaction surveillance system further. In the present study, the effectiveness of in-house premedication regimen with systemic corticosteroid and/or antihistamine on the occurrence and severity of immediate RCM hypersensitivity reactions was analysed. The impact of our novel RCM hypersensitivity surveillance and automatic recommending system for premedication on the management of hospitalised patients with a history of RCM hypersensitivity reactions in our general hospital was also assessed.

## Methods

### Previous drug hypersensitivity reaction surveillance system

The Asan Adverse Drug Reaction EMR Surveillance System (ADDRESS) has been operating in our hospital since March, 2003. Asan Medical Center is a university hospital in Seoul, Korea that operates 2291 licensed beds and admits approximately 100,000 patients annually. ADDRESS requires mandatory reporting of past DHRs of the patients at the time of admission. Any new cases of DHR that occur during the patient's current hospitalisation are entered on a voluntary basis. All DHRs recorded on the EMRs, including cases of RCM hypersensitivity, are reported to the adverse drug events monitoring team and, in severe cases, reviewed by allergy specialists [9].

After a DHR to a certain drug is reported, a pop-up banner warning regarding the culprit drug (including RCM) and its manifestations is generated and displayed on the EMR and the computerised physician order entry (CPOE) system whenever physicians access the patient's record. This pop-up banner thus encourages the physician who cares for patients with RCM hypersensitivity who require RCM administration to consider premedication with systemic corticosteroids, either alone or in combination with antihistamines. The decision to administer premedication is then made by the physician or radiologist. However, there was no intermediate step in this system that helped physicians to determine whether the patients needed premedication. A standard premedication protocol was also lacking. As a result, it remained difficult to control breakthrough RCM hypersensitivity reactions.

### Current RCM hypersensitivity reaction surveillance system

After ADDRESS was launched, we decided to reinforce it in 2009 by improving the comprehensive identification of RCM hypersensitivity including a classification of the previous RCM hypersensitivity reaction and ensuring that patients who experienced RCM hypersensitivity were managed more efficiently, as follows.

There were two major changes in the new system. First, the DHRs of the patient during the mandatory reporting at the time of admission are now subdivided into “RCM hypersensitivity reactions” and “DHRs other than RCM hypersensitivity”. Moreover, when a previous RCM hypersensitivity is reported, it is defined as an index reaction that is classified on the basis of its manifestations as being mild or severe (Table 1). Pruritus and localised urticaria are classified as mild reactions, while severe reactions are defined by the presence of one or more of the following systemic symptoms: angio-oedema, generalised urticaria, dyspnoea, bronchospasm, hypotension, and cardiopulmonary arrest.

**Table 1:** Classification of index and breakthrough reactions on the basis of symptoms and signs.

Reaction severity	Symptoms and signs
Mild	Pruritus, localised urticaria
Severe	Angio-oedema, generalised urticaria, dyspnoea, bronchospasm, hypotension, cardiopulmonary arrest

The second change was that premedication is recommended automatically by the CPOE system on the basis of the severity of the index reaction. Thus, when physicians insert an order for a computed tomography (CT) scan that requires the administration of RCM in a patient with a history of RCM hypersensitivity, the CPOE system recognises the entry of the order and then recommends in-house premedication regimens *via* a pop-up banner (Table 2). Depending on the severity of the index reaction, the in-house premedication regimens consist of systemic antihistamines and/or corticosteroids: patients with a mild index reaction receive chlorpheniramine 4 mg alone intravenously or intramuscularly 1 hour before RCM administration, while patients with severe index reactions receive intravenous hydrocortisone 200 mg plus chlorpheniramine 4 mg 1 hour before RCM is administered. Since these recommendations are not mandatoryand can change depending on the condition of the patient, all medical staff received special in-service education about RCM hypersensitivity premedication at the beginning of this project. The final decision to administer premedication is made at the discretion of the clinicians who order the CT scan.

**Table 2:** The standard premedication regimen used in the current system to prevent radiocontrast media (RCM) hypersensitivity.

Drug	Time and dose
Corticosteroid	Hydrocortisone 200 mg intravenously1 hour before RCM administration
Antihistamine	Chlorpheniramine 4 mg intravenously or intramuscularly1 hour before RCM administration

RCM: radiocontrast media.

Breakthrough reactions are also classified according to the criteria summarised in Table 1, and the RCM that were used and the management of breakthrough reactions are also mandatorily reported to our EMR system.

### Comparison of the previous and current RCM surveillance systems in terms of premedication use and outcome

Data over the same duration (12 months) were collected from the two systems, namely, between 1 July, 2008 and 30 June, 2009 for the previous system and between 1 March, 2010 and 28 February, 2011 for the current system. During these two study periods, only low osmolality RCM were used in our hospital. The index reaction severity, premedication regimen type, and occurrence of breakthrough reactions in all patients with a history of RCM hypersensitivity who were admitted during the two study periods were obtained from the EMR system. A breakthrough reaction was defined as an immediate hypersensitivity reaction after a CT scan using RCM in a patient who has been premedicated with corticosteroid and/or antihistamines.

The profiles of the two temporal cohorts, including their age and sex, were compared. The two systems were then compared in terms of the premedication regimen that was used, the rates of breakthrough reactions relative to the severity of the index reaction, and the effectiveness of premedication in terms of breakthrough reaction rates.

The chi-square test and Fisher's Exact test were used to analyse categorical variables. A *p*-value <0.05 was considered to be statistically significant.

### Ethics

This study was approved by the Internal Review Board and Ethics Committee of Asan Medical Center and they decided that any informed consent was not needed in this study, because this study was performed by retrospective chart review and the patients' identifications were all deleted.

## Results

### Baseline characteristics of the two RCM-hypersensitive temporal cohorts

In the 12-month study periods of the previous and current systems, there were 31356 CT scans requiring RCM (on 17933 patients) and 39186 ones (on 21106 patients) in total, respectively. Three hundred sixty two and 403 patients with a history of RCM hypersensitivity were admitted and checked by CT scans during the 12-month study periods when the previous and current systems were operating, respectively. The two cohorts did not differ significantly in terms of gender (60% and 55% were male, respectively). They also did not differ in terms of age: of the group managed under the previous system, 3%, 67% and 30% were <20, 20–65 and >65 years old, respectively, while the corresponding frequencies for the group managed under the current system were 0%, 70% and 30%, respectively (Table 3).

**Table 3:** Comparison of the previous and current systems in terms of index reaction rates, premedication rates, and rates of breakthrough reaction.

	Previous system	Current system
	(N = 362)	(N = 403)
Demographic data		
Sex (M/F)	220/142	261/142
Age (years)		
<20	3	0
20–39	30	15
40–59	178	189
>60	151	199
Index reaction		
Severe reaction	48 (13%)	103 (26%)
Mild reaction	314 (87%)	300 (74%)
Premedication (%)	330 (91%)	384 (95%)
Antihistamine	278 (77%)	122 (30%)
Corticosteroid and antihistamine	52 (14%)	262 (65%)
Breakthrough reaction (%)	55 (15.2%)	27 (6.7%)
Mild reaction	46 (12.7%)	24 (6%)
Severe reaction	9 (2.5%)	3 (0.7%)

Of the 362 previous system patients, 13% and 87% had severe and mild index reactions, respectively. Of the 403 current system patients, 26% and 74% had severe and mild index reactions, respectively. This difference was statistically significant (*P*-value = 0.031) (Table 3).

The two groups also did not differ in terms of the RCM that were used during the CT scans (*P*-value = 0.078): 47%, 37%, 9%, 4%, 1% and 2% of the group managed under the previous system were administered with Ultravist™ (Iopomide), Optiray™ (Ioversol), Omnipaque™ (Iohexol), Iopamiro™ (Iopamidol), Pamiray™ (Iopamidol) and others, respectively, while the corresponding frequencies for the group managed under the current system were 38%, 42%, 5%, 3%, 10% and 2%, respectively.

### Comparison of the previous and current systems in terms of premedication and breakthrough reaction rates

**Figure 1:**
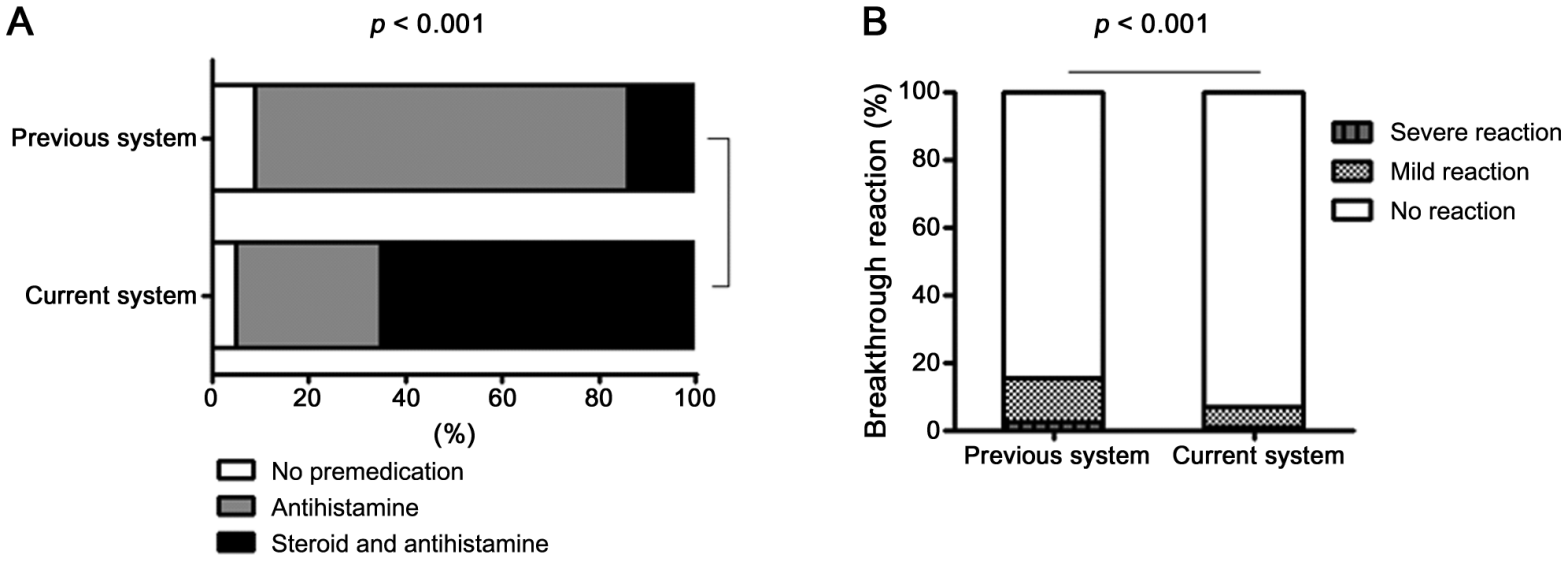
Comparison of the previous and current systems in terms of premedication and breakthrough reaction rates in all patients with index reactions.

In the previous system, 330 of the 362 patients (91%) were pretreated with systemic antihistamines and/or corticosteroids, while in the current system, 384 of the 403 patients (95%) were pretreated in this manner. However, only 52 of the previous system patients (14%) received both systemic corticosteroid and antihistamine. In contrast, 263 of the current system patients (65%) were pretreated with both systemic corticosteroid and antihistamine (*P*-value<0.001) (Table 3, Fig. 1A).

The current system was associated with a significant reduction in the incidence of the breakthrough reactions after premedication: there were 27 (6.7%) breakthrough reactions in the current system cohort compared to 55 (15.2%) reactions in the previous system cohort (*P*-value<0.001) (Table 3, Fig. 1B). The current system was also associated with a reduction in the incidences of both severe and mild breakthrough reactions: 0.7% and 6% of the current system cohort had a severe and mild breakthrough reaction, respectively, whereas 2.5% and 12.7% of the previous system cohort had a severe and mild breakthrough reaction, respectively, although severe reactions did not achieve statistical significance. .

### Analysis of the premedication and breakthrough reaction rates between the two systems according to the severity of the index reaction

**Figure 2:**
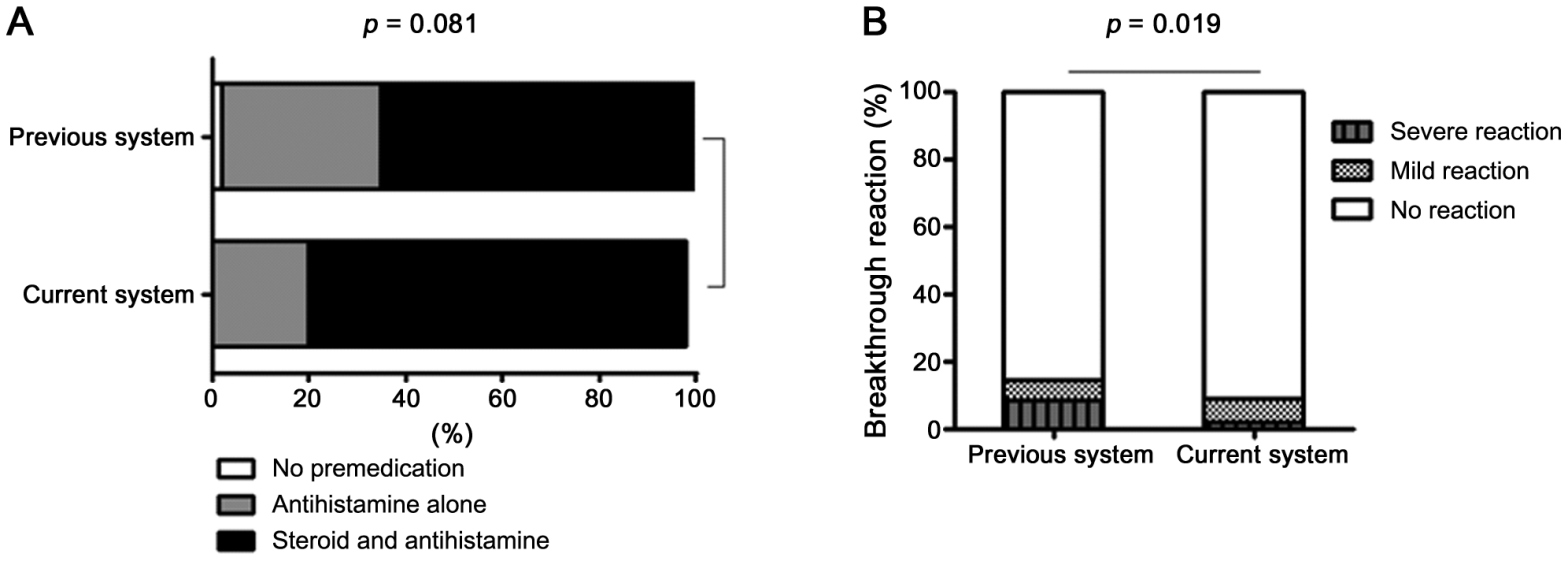
Comparison of the previous and current systems in terms of premedication and breakthrough reaction rates in patients with a severe index reaction.

In the previous system cohort 48 patients had a severe index reaction. Of these, 31 (65%) and 16 (33%) patients were pretreated before a CT scan using RCM with corticosteroid plus antihistamine and antihistamine alone, respectively. The remaining patient (2%) did not receive any premedication. By contrast, in the current system cohort, there were 103 patients who had a severe index reaction, of whom 80 (78%) and 23 (22%) were pretreated with corticosteroid plus antihistamine and antihistamine alone, respectively. None did not receive any premedication (*P*-value = 0.081). Thus, in the current system, systemic corticosteroids were used more frequently to premedicate patients who had a severe index reaction than in the previous system (Fig. 2A).

Of the 48 previous system patients who had a severe index reaction, seven (14.5%) had a breakthrough reaction. In contrast, of the 103 current system patients who had had a severe index reaction, nine (9%) had a breakthrough reaction (*P*-value = 0.19) (Fig. 2B). The current system was also associated with less severe breakthrough reactions in patients who had a severe index reaction: in the previous system, 8.3% and 6.2% of the severe index cases had a severe and mild breakthrough reaction, respectively, while the corresponding frequencies for the current system were 2% and 7%, respectively. However, this trend did not achieve statistical significance.

**Figure 3:**
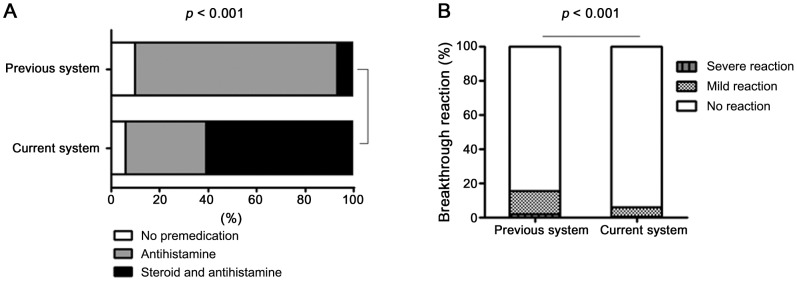
Comparison of the previous and current systems in terms of premedication and breakthrough reaction rates in patients with a mild index reaction.

In the previous system cohort, 314 patients had a mild index reaction. Of these, 21 (7%) and 262 (83%) were pretreated with corticosteroid plus antihistamine and antihistamine alone, respectively, and 31 (10%) were not pretreated. By contrast, in the current system cohort, 300 patients had a mild index reaction. Of these, 182 (61%) and 99 (33%) were pretreated with corticosteroid plus antihistamine and antihistamine alone, respectively and 19 (6%) were not pretreated (*P*-value<0.001) (Fig. 3A). Thus, in the current system, systemic corticosteroids were used significantly more frequently to treat patients with mild index RCM hypersensitivity than in the previous system.

Of the 314 previous system patients with a mild index reaction, 48 (15.3%) had a breakthrough reaction. In contrast, of the 300 current system patients with a mild index reaction, only 18 (6%) had a breakthrough reaction (*P*-value<0.001) (Fig. 3B). Thus, the current system was associated with a significant reduction in breakthrough reactions in patients who had a mild index reaction. Moreover, the breakthrough reactions of these patients were significantly less likely to be severe in the current system: in the previous system, 1.6% and 13.7% had a severe and a mild reaction, respectively, while the corresponding rates for the current system were 0.3% and 5.7%, respectively.

### Relationship between the index and breakthrough reactions under the current system

**Figure 4:**
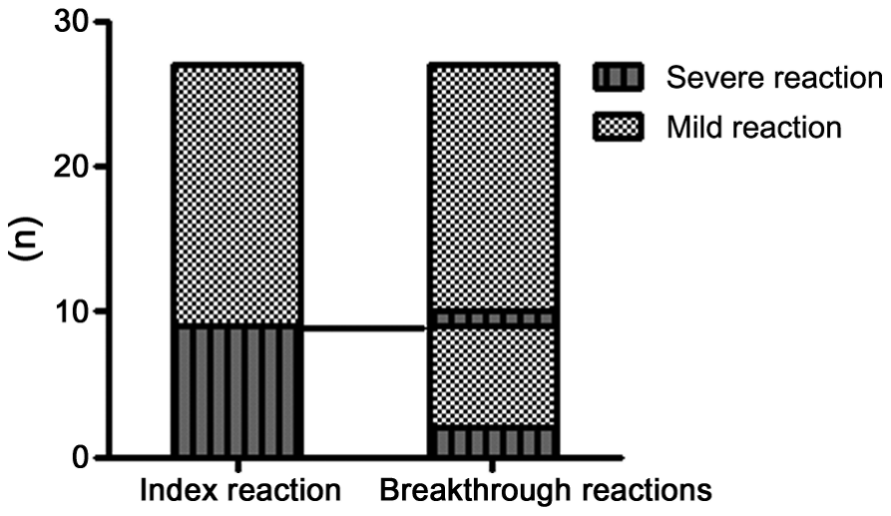
Relationship between the severity of the index and breakthrough reactions under the current system.

Under the current system, 27 breakthrough reactions (24 mild, 3 severe) were reported. Analysis of the severity of the index and breakthrough reactions revealed that in the 18 patients with a mild index reaction, the breakthrough reactions were similarly mild in 17 (94%). Only one patient (4%) had a severe breakthrough reaction. The nine patients who had a severe index reaction were slightly more likely to have a severe breakthrough reaction (2, 22%) than the patients who had a mild index reaction, although this trend did not achieve statistical significance (*P*-value = 0.25) (Fig. 4). However, most of the breakthrough reactions of the patients with severe index reactions were mild.

The severity of the breakthrough reactions under the current system was not affected by a change in RCM relative to the RCM that was used in the index reaction (*P*-value = 0.07). The interval between the index and breakthrough reactions also did not affect breakthrough reaction severity significantly (*P*-value = 0.56) (Table 4).

**Table 4:** Effect of changing the radiocontrast media (relative to the index reaction) and the interval between the index and breakthrough reactions on the severity of breakthrough reactions under the current system.

Parameter	Change in reaction severity*		
	Decreased	Same	Increased
Number of patients	4	12	1
Demographic data			
Sex (M/F)	2/2	10/2	0/1
Age (years)			
<20	0	0	0
20–39	0	0	1
40–59	4	8	0
>60	0	4	0
Number of CT scan	8	18	1
Radiocontrast media			
Same (n = 7)	4 (57%)	3 (43%)	-
Different (n = 20)	4 (20%)	15 (75%)	1 (5%)
Interval between index and breakthrough reactions (months)			
0–6	7	13	1
6–12	1	4	-
>12	-	1	-

* Severity of the breakthrough reaction compared to the severity of the index reaction.

Eight patients had multiple breakthrough reactions. Six of these had two additional breakthrough reactions after the index reaction and the remaining two patients had three additional breakthrough reactions. None of breakthrough reactions of the eight patients were more severe than the index reaction (Table 5).

**Table 5:** Premedication and severity of the radiocontrast media hypersensitivity reactions in eight patients with multiple breakthrough reactions.

Patient number (Sex/Age)	Index reaction	Breakthrough reaction 1(Premedication)	Breakthrough reaction 2(Premedication)	Breakthrough reaction 3(Premedication)
1 (M/51)	Mild	Mild(corticosteroid+peniramine)	Mild(corticosteroid+peniramine)	N/A
2 (F/57)	Mild	Mild(corticosteroid+peniramine)	Mild(corticosteroid+peniramine)	N/A
3 (M/54)	Mild	Mild(peniramine)	Mild(peniramine)	N/A
4 (M/51)	Mild	Mild(peniramine)	Mild(corticosteroid+peniramine)	N/A
5 (M/46)	Severe	Mild(peniramine)	Mild(peniramine)	N/A
6 (F/58)	Severe	Mild(peniramine)	Mild(peniramine)	N/A
7 (72/F)	Mild	Mild(peniramine)	Mild(peniramine)	Mild(peniramine)
8 (58/M)	Severe	Mild(corticosteroid+peniramine)	Mild(corticosteroid+peniramine)	Mild(corticosteroid+peniramine)

N/A = not applicable.

## Discussion

The present study showed that our novel RCM hypersensitivity surveillance and automatic recommending system for prescription effectively improved premedication rates in a general hospital. It also showed that premedication with systemic corticosteroid and/or antihistamine significantly reduced immediate-type breakthrough reactions in patients who had had RCM hypersensitivity reactions even mild index reactions.

To our knowledge, our system is the first computerised RCM hypersensitivity surveillance and automatic recommending system in the world. The present study showed that severe index reaction reports rose from 13% in the previous system to 26% in the present system. One of the avoidable errors in dealing with hypersensitivity reactions to RCM is “Failure to get adequate pre-examination clinical assessment” [10]. In fact, our mandatory computerised reporting system has improved the detection rate of patients who had prior RCM hypersensitivity reactions. This confirms the findings of other studies showing that computerised reporting systems identify adverse drug events effectively [11]. Moreover, the automatic recommendation of standard premedication relative to the severity of the index reaction in our system helps the decision-making of the clinician regarding the premedication of patients who have a history of RCM hypersensitivity and require a CT scan with RCM. Notably, only 78% of patients with severe index reactions were pretreated with corticosteroid and antihistamine. It is possible that some patients were not premedicated with systemic corticosteroid because of severe infection, viral hepatitis and poor glycemic control (among other possibilities) during admission.

Although the increased use of non-ionic, low osmolality RCM is associated with a reduced incidence of RCM hypersensitivity reactions, premedication is still widely used in clinical practice, especially with patients who have had prior RCM hypersensitivity reactions [6]. Over the last few decades, many premedication protocols have been suggested; these regimens vary considerably [2], [12], [13]. In addition, the effectiveness of premedication in preventing RCM hypersensitivity reactions in patients with a history of RCM hypersensitivity remains poorly understood [14]. It has been reported that between 10% and 18% of corticosteroid-premedicated patients who receive low osmolality contrast media have breakthrough reactions [15], [16]. In the present study, a relatively simple premedication protocol served as the in-house premedication regimen. In fact, Lasser and colleagues demonstrated that an oral dose of a steroid given two hours before contrast material administration was no better in preventing subsequent reactions to high-osmolality contrast material than was placebo [17]. However, the overall rate of breakthrough reaction was reduced from 15.2% to 6.7% in our current system, which indicates that our simple premedication works in reducing breakthrough reactions in cases of use of non-ionic, low osmolality of RCM. We think that our protocol can be applied to other general hospitals for the simplicity and convenience.

Different premedication protocols were recommended by the CPOE system depending on the severity of the index reaction in this study: patients with a mild index reaction only received antihistamine 1 hour before RCM administration, whereas patients with severe index reactions received both corticosteroid and antihistamine. The reason is that there is no standard regimen for premedication in cases the previous RCM hypersensitivity was just mild reaction. However, the data analysis revealed that of the 300 patients who had a history of mild index reaction in the current system, 182 (61%) were premedicated with both corticosteroid and antihistamine. Indeed, the corticosteroid premedication rate of patients with mild index reactions increased significantly by about eightfold under the current system. Thus, it appears that even when the index reaction was mild, the clinicians tended to choose a combination of corticosteroid and antihistamine for premedication rather than antihistamine alone. Since the breakthrough reactions of the patients with mild index reactions were remarkably reduced, it may be that higher corticosteroid premedication rates in even mild index reaction cases may help to reduce the incidence of breakthrough reactions overall. However, it is not clear whether the tendency to premedicate with corticosteroid directly caused the drop in breakthrough reaction incidence in mild index reactions. Further randomized controlled studies would be needed to show this.

In the present study, 27 patients experienced breakthrough reactions. However, only one (5%) exhibited higher severity relative to the index reaction (i.e. mild to severe). Moreover, the patients who had multiple breakthrough reactions during the study period did not exhibit increasing severity; indeed, the breakthrough reactions remained less severe than the index reactions, probably because of premedication. These findings are consistent with the previously published data of Davenport *et al.*, which showed that breakthrough reactions induced by low osmolarity contrast media were usually similar (81%) to the index reaction; the breakthrough reactions were more severe in only 8% of cases [16]. However, physicians should be aware of the possibility that premedication can fail, even when the index reaction is mild.

There were some limitations in this study. First, the definition of RCM hypersensitivity severity was too simply defined for the convenience to the real users of the reporting system; this could affect the effectiveness of index reaction severity-related premedication. However, we excluded localized redness or itching at the injection site to differentiate from pharmacological side effects in our study. In addition, although there may be difference between generalized urticaria treatable with antihistamines and more severe anaphylaxis eventually needing epinephrine treatment, however, we considered any angioedema or generalized urticaria as severe reaction because it could be developed to anaphylaxis. Strictly speaking, moderate reactions might be included in severe reactions in our study. Second, specific risk factors of breakthrough reactions, including allergic work-ups, could not be identified in this study because this was a retrospective study for only the effectiveness of premedication *via* our new system. It may also be useful to determine whether RCM hypersensitivity can be predicted by conducting a skin test [18]. Indeed, our results showed a significant effect on mild reactions, but less on severe reactions. This may indicate that severe reactions may be specific allergic reactions not easily treated. Here skin tests might have been helpful to exclude allergy not responsive to pretreatment.

In conclusion, the present study showed premedication with corticosteroid and/or antihistamine, which was increased by our novel automatic prescription system, significantly reduced breakthrough reactions in patients with a history of RCM hypersensitivity.
